# *Schistosoma japonicum* infection-mediated downregulation of lncRNA Malat1 contributes to schistosomiasis hepatic fibrosis by the Malat1/miR-96/Smad7 pathway

**DOI:** 10.1186/s13071-024-06499-9

**Published:** 2024-10-03

**Authors:** Pengyue Jiang, Shengyu Ye, Xiaobin Fan, Yini Tian, Dongmei Zhang, Weiqing Pan

**Affiliations:** 1 Department of Tropical Diseases, Naval Medical University, Shanghai, China; 2 Institute for Infectious Diseases and Vaccine Development, Tongji University School of Medicine, Shanghai, China

**Keywords:** Malat1, miRNA-96, Smad7, *Schistosomajaponicum*, Schistosomiasis hepatic fibrosis

## Abstract

**Background:**

*Schistosoma japonicum* infection causes hepatic fibrosis, a primary cause of morbidity and mortality associated with the disease, and effective treatments are still lacking. Long non-coding RNAs (lncRNAs) have been implicated in the pathogenic process of various tissue fibroses. However, the role of lncRNAs in schistosomiasis hepatic fibrosis (HF) is poorly understood. Understanding the role of lncRNAs in schistosomiasis HF will enhance knowledge of disease processes and aid in the discovery of therapeutic targets and diagnostic biomarkers.

**Methods:**

Differentially expressed lncRNA profiles in primary hepatic stellate cells (HSCs) of mice infected with *S. japonicum* were identified using high-throughput lncRNA sequencing. Primary HSCs were isolated from infected mice using collagenase digestion and density-gradient centrifugation, cultured in DMEM with 10% fetal bovine serum. Dual-luciferase reporter assays, nuclear cytoplasm fractionation and RIP assays were employed to assess the relationship between Malat1 and miRNA-96. Malat1 lentivirus and ASO-Malat1 were constructed for forced expression and downregulated expression of Malat1. The Malat1-KO mouse was constructed by CRISPR/Cas9 technology. Pathological features of the liver were evaluated by hematoxylin-eosin (HE), Masson’s trichrome staining and immunohistochemistry (IHC). The expression levels of fibrosis-related genes were determined by quantitative real-time PCR (qRT-PCR) and Western blot.

**Results:**

A total of 1561 differentially expressed lncRNAs were identified between infected and uninfected primary HSCs. Among the top altered lncRNAs, the downregulated Malat1 was observed in infected HSCs and verified by qPCR. Treatment of infected mice with praziquantel (PZQ) significantly increased the Malat1 expression. Elevated Malat1 expression in infected primary HSC reduced the expressions of profibrogenic genes, whereas Malat1 knockdown had the opposite effect. Moreover, Malat1 was found to interact with miR-96, a profibrotic miRNA, by targeting Smad7. Forced Malat1 expression reduced miR-96 levels in infected primary HSCs, attenuating fibrogenesis and showing negative correlation between Malat1 expression and the expression levels of miR-96 and profibrogenic genes α-SMA and Col1α1. Notably, in Malat1-KO mice, knockout of Malat1 aggravates schistosomiasis HF, while restored Malat1 expression in the infected HSCs reduced the expression of profibrogenic genes.

**Conclusions:**

We demonstrate that lncRNA is involved in regulation of schistosomiasis HF. Elevated lncRNA Malat1 expression in infected HSCs reduces fibrosis via the Malat1/miR-96/Smad7 pathway, thus providing a novel therapeutic target for schistosomiasis HF. Furthermore, Malat1 expression is sensitive to PZQ treatment, thus offering a potential biomarker for assessing the response to chemotherapy.

**Graphical abstract:**

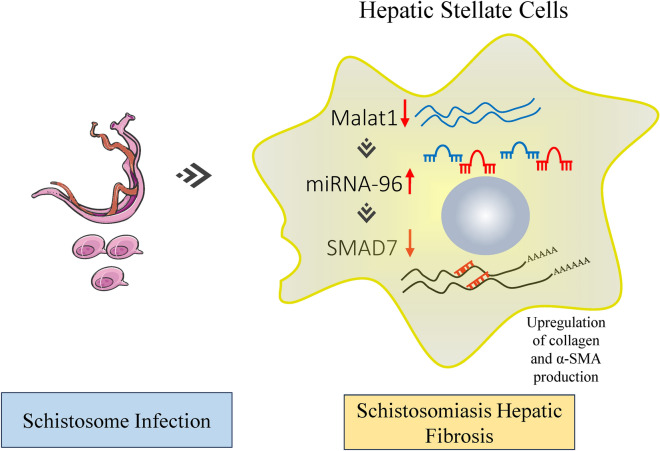

**Supplementary Information:**

The online version contains supplementary material available at 10.1186/s13071-024-06499-9.

## Background

Schistosomiasis is a serious parasitic disease endemic to tropical and subtropical regions, with approximately 779 million people at a risk of infection and 250 million people infected worldwide [1, 2]. Schistosomiasis japonica, primarily endemic to China and the Philippines, caused by the genus *Schistosoma japonicum*, one of six *Schistosoma* species that can infect humans. Schistosomiasis japonicum has a complex life cycle. In the definitive human host, the adult female in the mesenteric veins of the bowel produces numerous eggs, some of which are excreted in the feces through the intestinal wall, while the others are transported to the liver via portal veins. The trapped eggs in the liver secrete soluble antigens, inducing the formation of granulomas around eggs, containing various inflammatory cells such as neutrophils [3, 4]. This results in severe periportal fibrosis with collagen deposition around the portal vein [5], leading to portal hypertension.

Hepatic stellate cells (HSCs), constituent cells of the liver, reside in the space between liver sinusoidal endothelial cells and hepatocytes and function as vitamin A storage [6]. Under physiological conditions, HSCs exist as quiescent cells. However, in response to liver injury, the quiescent HSCs can be activated, becoming proliferative and expressing profibrogenic genes [7]. The activated HSCs secrete excess extracellular matrix (ECM) in the liver, leading to hepatic fibrosis. In the schistosomiasis HF, previous studies have demonstrated the presence of activated HSC in the egg granulomas [8], indicating that activation of HSCs is essential for schistosomiasis HF. Transforming growth factor 1 (TGF-β1) is a classic modulator for the activation of HSCs via the TGF-β1/SMAD pathway. However, in schistosomiasis HF, in addition to TGF-β1, IL-13 is also a critical profibrotic factor, exerting its profibrogenic effects through activation of distinct SMAD proteins [9], indicating a more complex regulatory mechanism.

Accumulating evidence indicates that *S. japonicum* infection induces deregulation of microRNAs (miRNAs) in the host, associated with schistosomiasis HF. Our previous studies have shown that *S. japonicum* infection significantly elevates miR-21 and miR-96, which promote hepatic fibrosis by targeting Smad7 [10, 11]. The efficient and sustained inhibition of these altered miRNAs in the liver alleviated hepatic fibrosis and protected the host from lethal schistosomiasis via attenuating hepatic fibrosis [10, 11]. Long non-coding RNA is usually defined as an RNA transcript longer than 200 nucleotides that does not have protein-coding capacity or can encode short peptides [12]. A growing number of studies demonstrate that lncRNAs are involved in human diseases such as cancers [13–15] and fibrosis [16, 17]. Upregulation of a lncRNA designated as a pro-fibrotic lncRNA (PFL) promoted cardiac fibrosis by acting as a competing endogenous RNA of let-7d [17]. In addition, Malat1 was involved in the regulation of fibrotic diseases in various organs [18–20]. Recently, numerous lncRNAs have been identified in the schistosome genome [21]. Although host miRNA has been shown to be involved in schistosomiasis HF, the role of lncRNA in this regard remains unclear. In this study, we performed screening of altered lncRNAs in the HSCs of mice infected with *S. japonicum* and identified 1561 differentially expressed lncRNAs. Notably, Malat1 was significantly downregulated in the schistosome-infected mice. Gain- or loss-of-function experiments in vitro and in Malat1-KO mouse models in vivo revealed its suppressive effect on the progression of schistosomiasis HF through Malat1/miR-96/Smad7 axis.

## Methods

### Mice and schistosomiasis model

Adult wild-type male C57BL/6 mice, aged 6–8 weeks, were purchased from the Experimental Animal Center of the Naval Medical University (Shanghai, China) and raised in specific pathogen-free conditions. All animal experiments were conducted in accordance with the Guidelines for the Care and Use of Laboratory Animals of the National Institutes of Health, and the study received approval from the Animal Ethics Committee of the Naval Medical University (approval number FYXK [Shanghai] 2014–0003). Malat1-KO heterozygote mice on a C57BL/6 background were purchased from the Shanghai Model Organisms Center, Inc. (#NM-KO-201701), and were bred to obtain sufficient numbers of homozygotes for subsequent experiments. Schistosomiasis models were established by percutaneously exposing mice to 30 *S. japonicum* cercariae shed from laboratory-cultured snails (*Oncomelania hupensis*) obtained from the National Institute of Parasitic Disease, Chinese Center for Disease Control and Prevention (Shanghai, China).

### Isolation and culture of primary HSCs

Primary HSCs were isolated from schistosomiasis mouse models or normal mice using collagenase digestion and density-gradient centrifugation, followed by purification with magnetic CD11b antibody beads (Miltenyi, Germany) by negative selection to exclude Kupffer cells. HSCs were plated on plastic dishes in DMEM containing 10% fetal bovine serum (Gibco, #10099–141), 1% penicillin-streptomycin (100 mg/ml) and 4 mM l-glutamine (100 IU/ml) and cultured in an incubator (37 °C, 5% CO_2_, 95% humidity).

### lncRNA sequencing and analysis

RNA samples from primary HSCs of *S. japonicum*-infected and control mouse livers were prepared using TRIzol reagent for lncRNA-seq (three biological replicates for each group). Total RNA was quantified using a NanoDrop-2000 spectrophotometer (Thermo Fisher Scientific, Wilmington, DE), and RNA integrity was assessed using the RNA Nano 6000 Assay Kit of the Agilent Bioanalyzer 2100 System (Agilent Technologies, CA, USA). Ribosomal RNA was removed using the Ribo-Zero rRNA Removal Kit (Epicentre, Madison, WI, USA) to enable accurate lncRNA analysis. Sequencing libraries were generated using the NEBNext® Ultra™ Directional RNA Library Prep Kit for Illumina^®^ (NEB, USA) following the manufacturer's recommendations, and the libraries were sequenced on an Illumina platform, generating reads. Only reads with a perfect match were further analyzed and annotated based on the reference genome. HISAT2 software was used to map reads to the reference genome. The lncRNA sequencing and bioinformatic analysis were conducted by BioMarker Biotech Co., Ltd. (Beijing, China).

### RNA preparation and quantitative real-time PCR

Total RNA was extracted from liver tissue, isolated HSCs, or cell lines using TRIzol reagent (Sigma-Aldrich, #T9424), and RT reactions were performed according to the instructions provided with the HiScript III RT SuperMix for qPCR (+ gDNA wiper) kit (Vazyme #R323) or miRNA 1st Strand cDNA Synthesis Kit (by stem-loop) (Vazyme #MR101). Assays to quantify miRNA-96, lncRNA, or mRNA were performed using ChamQ™ Universal SYBR^®^ qPCR Master Mix (Vazyme #Q711, Nanjing, China) on a QuantStudio 6 real-time PCR system (Applied Biosystems). U6 small nuclear RNA and GAPDH were used as internal controls for miRNA and mRNA/lncRNA, respectively, and the relative expression was calculated using the 2^−ΔΔCt^ method [22]. The primers used in this study are listed in Table S1.

### Cell culture and transfection

Mouse primary HSCs, the mouse immortalized HSC cell line JS1, and the human HSC line LX-2 were cultured in DMEM mixed with 10% fetal bovine serum, 1% antibiotics, and 4 mM l-glutamine in an incubator (37 °C, 5% CO_2_, 95% humidity). When cell density reached 70% confluence in a 12-well plate, cells were transfected with miRNA-96 mimics, inhibitor miRNA-96, ASO-Malat1, siRNA-Malat1, relevant plasmids, or corresponding negative controls using Lipofectamine 3000 (Life Technology, #L3000-008) according to the recommended protocol. Cells were collected for detection 48 h post-transfection. The ASO compound was purchased from RiboBio Co. (Guangzhou, China), and other chemicals were synthesized and purified by GenePharma (Shanghai, China). The detailed sequences of the mimics, inhibitors, and siRNAs are shown in Table S2.

### Lentivirus (LV) construction and infection

The expression plasmid for Malat1 was constructed by PCR and inserted into the pCDH-CMV-EF1-Puro vector. LV-Malat1 or LV-Scramble was produced by co-transfecting HEK293T cells cultured in 10-cm dishes with pCDH-CMV-EF1-Puro-Malat1 or empty vectors and packaging plasmids (psPAX.2 and pMD2.G) at a ratio of 4:3:1. After 48 h of transfection, the lentivirus supernatant was filtered through a 0.45-μm filter, followed by infection of cells with 10 μg/ml polybrene. One day after infection, the medium was replaced with fresh medium and cultured for 48 h before cells were collected for further analysis.

### Dual-luciferase reporter assays

The binding site between Malat1 and miRNA-96 was predicted using Starbase (https://starbase.sysu.edu.cn/). The fragments of Malat1 wild-type (wt) and mutant (mt) miRNA-96 binding sites were cloned into the pmirGLO dual-luciferase plasmid (Promega, USA) to construct Malat1-WT/Mut. Constructs were co-transfected with miRNA-96 or NC mimics into HEK-293 T cells for 48 h. Dual-luciferase reporter assay system (Promega) was used to assess luciferase activity.

### Nuclear cytoplasm fractionation

Cytoplasmic and Nuclear RNA Purification Kit (Norgen, Canada) was used to detect cellular accumulation of Malat1 according to the manufacturer's instructions. Lysed cells in cell fractionation buffer were collected for centrifugation and RNA isolation. Malat1 content in cell cytoplasm and nucleus was detected by RT-qPCR, with Gapdh and U6 used as cytoplasm and nucleus controls, respectively.

### RNA immunoprecipitation (RIP)

MagnaRIP™ RNA-Binding Protein Immunoprecipitation Kit (Millipore, Bedford, USA) was used for RIP assay according to standard procedures. Cell lysates collected from RIP lysis buffer were conjugated with Ago2 (CST, #2897) antibody or control IgG antibody and magnetic beads. The immunoprecipitates were analyzed via RT-qPCR.

### Western blot

Cell proteins were extracted by washing cells with PBS (pH 7.4) and lysing with RIPA Lysis buffer (Beyotime, China) supplemented with a Protease and Phosphatase Inhibitor Cocktail (Thermo Scientific #78440). Protein concentration in cell lysates was quantified using the BCA method (Beyotime, China). A total of 20–30 μg of protein was loaded on a 10–16% polyacrylamide gel per sample. Proteins were transferred onto polyvinylidene fluoride (PVDF; Millipore, USA) membranes and incubated with primary antibodies, including anti-α-SMA (abcam, #ab7817), anti-Col1α1 (CST, #72026), anti-Smad7 (Santa Cruz, #sc-365846) and anti-Gapdh (Proteintech, #10494–1-AP), overnight at 4 °C. Membranes were then incubated with secondary antibodies (Abcam) for 1 h at room temperature, and an enhanced chemiluminescent substrate kit (Millipore) was used to visualize the proteins.

### Pathological features of the liver

After anesthetizing mice, the right liver lobes were isolated and fixed with 4% paraformaldehyde. Processed liver sections were subjected to hematoxylin-eosin (HE), Masson’s trichrome staining and IHC analysis. Hydroxyproline Content Assay Kit was used to detect the hydroxyproline content of liver tissues according to the kit’s instructions (Nanjing Jiancheng, China, #A030-2). Parasites were counted in a sterile petri dish containing PBS medium, and a portion of the liver tissue was digested overnight in 4% potassium hydroxide. Eggs were then counted and calculated as 10^4^ eggs per gram of liver tissue.

### Statistical analysis

All results are reported as means ± standard deviations and compared between groups using two-tailed Student’s t-test or one-way ANOVA. Data were considered statistically significant at *P* < 0.05.

## Results

### *Schistosoma japonicum* infection-mediated downregulation of Malat1 in HSC.

**Fig. 1:**
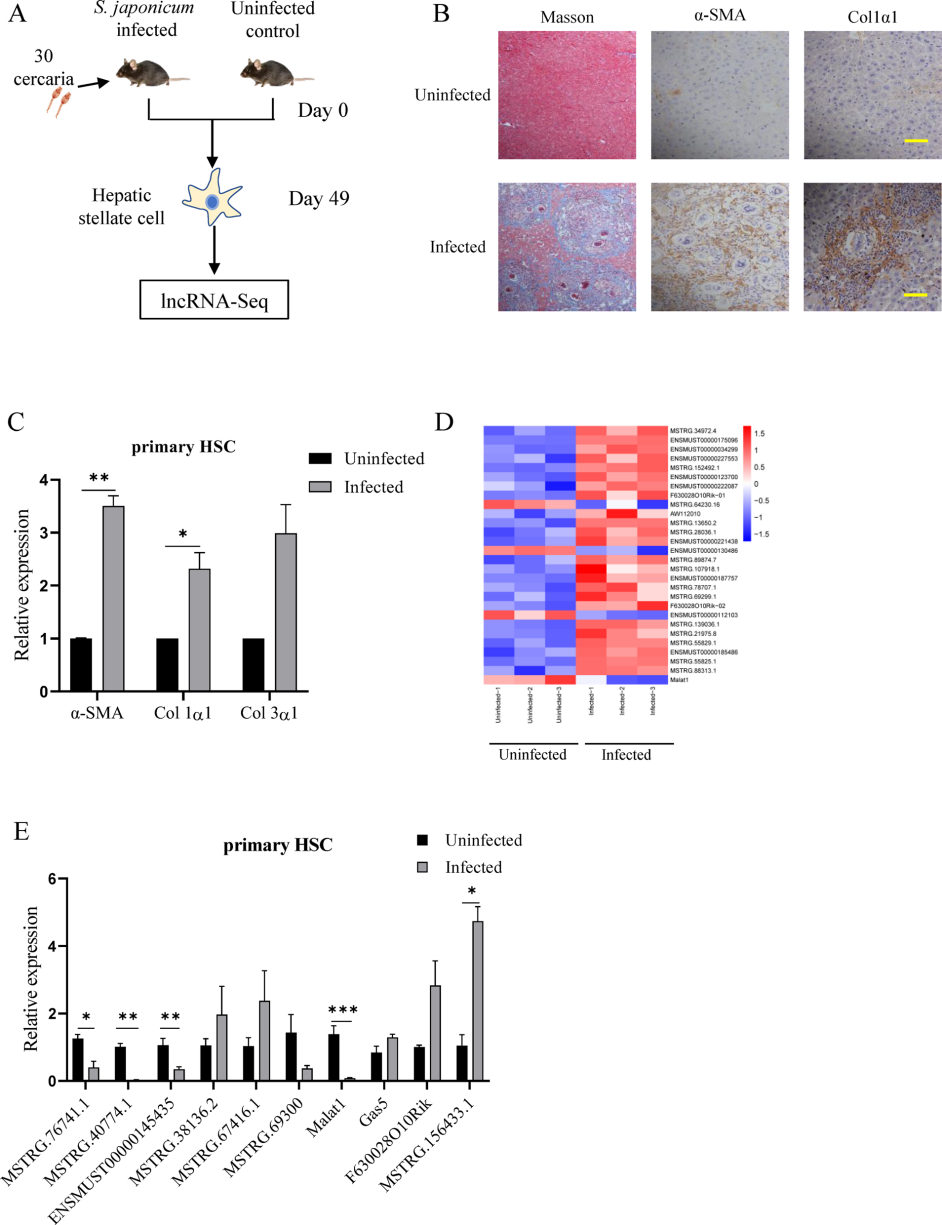
*Schistosoma japonicum* infection-mediated downregulation of Malat1 in HSCs. **A** Schematic diagram of experimental design. Primary HSCs isolated from *S. japonicum*-infected and normal control male C57BL/6 mice at day 49 after infection were used for lncRNA-seq, three samples each group. **B** Representative Masson's trichrome staining and IHC for α-SMA and Col1α1 in the liver sections of mice (scale bars: 50 μm). **C** Expression of Col1α1, Col3α1 and α-SMA mRNA in HSCs from infected and uninfected primary HSCs (*n* = 3). **D** Heatmap of the altered expression lncRNAs according to fold-change value. **E** Expression levels of 10 representative lncRNAs was validated in infected and uninfected HSCs by qPCR (*n* = 5). *p < 0.05, ** *p* < 0.01, *** *p* < 0.001

To explore the differentially expressed lncRNAs profiles in primary HSCs between *S. japonicum* infected and uninfected mice, the liver tissues were collected from the infected mice at day 49 post infection when significant liver fibrosis has been formed, as detected by Masson’s trichrome staining and immunohistochemistry, as manifested by more collagen depositions and larger positive area of α-SMA and Col1α1 in the infected liver compared to the uninfected mice (Fig. 1A, B). Moreover, the primary HSCs isolated from the infected mice revealed elevated expression of the fibrosis-related genes encoding α-SMA and Col1α1 by qPCR compared with the uninfected mice (Fig. 1C), indicating that HSCs were already activated. We then performed lncRNA-seq of the isolated HSCs and identified 1561 differentially expressed lncRNAs between infected and uninfected primary HSCs. Among them, 935 were upregulated and 626 downregulated. Heat map analyses of the altered lncRNAs with top fold-change values showed Malat1 was significantly downregulated in infected HSCs (Fig. 1D). Additionally, in situ hybridization on the liver, using Digoxin-labeled Malat1 probe, revealed that Malat1 was significantly downregulated in the liver granulomatous tissue of infected mice, which was consistent with the sequencing results (Figure S1). Furthermore, we verified the expression of 10 lncRNAs that showed differential expression in the sequencing data by qPCR using an additional batch of primary HSC samples and showed that most lncRNA expression, including the Malat1, was consistent with the sequencing data (Fig. 1E). Collectively, our data indicated that *S. japonicum* infection can regulate lncRNA expression in HSCs, with significant downregulation of Malat1.

### Forced expression of Malat1 in infected HSCs inhibited the expression of profibrogenic genes

**Fig. 2:**
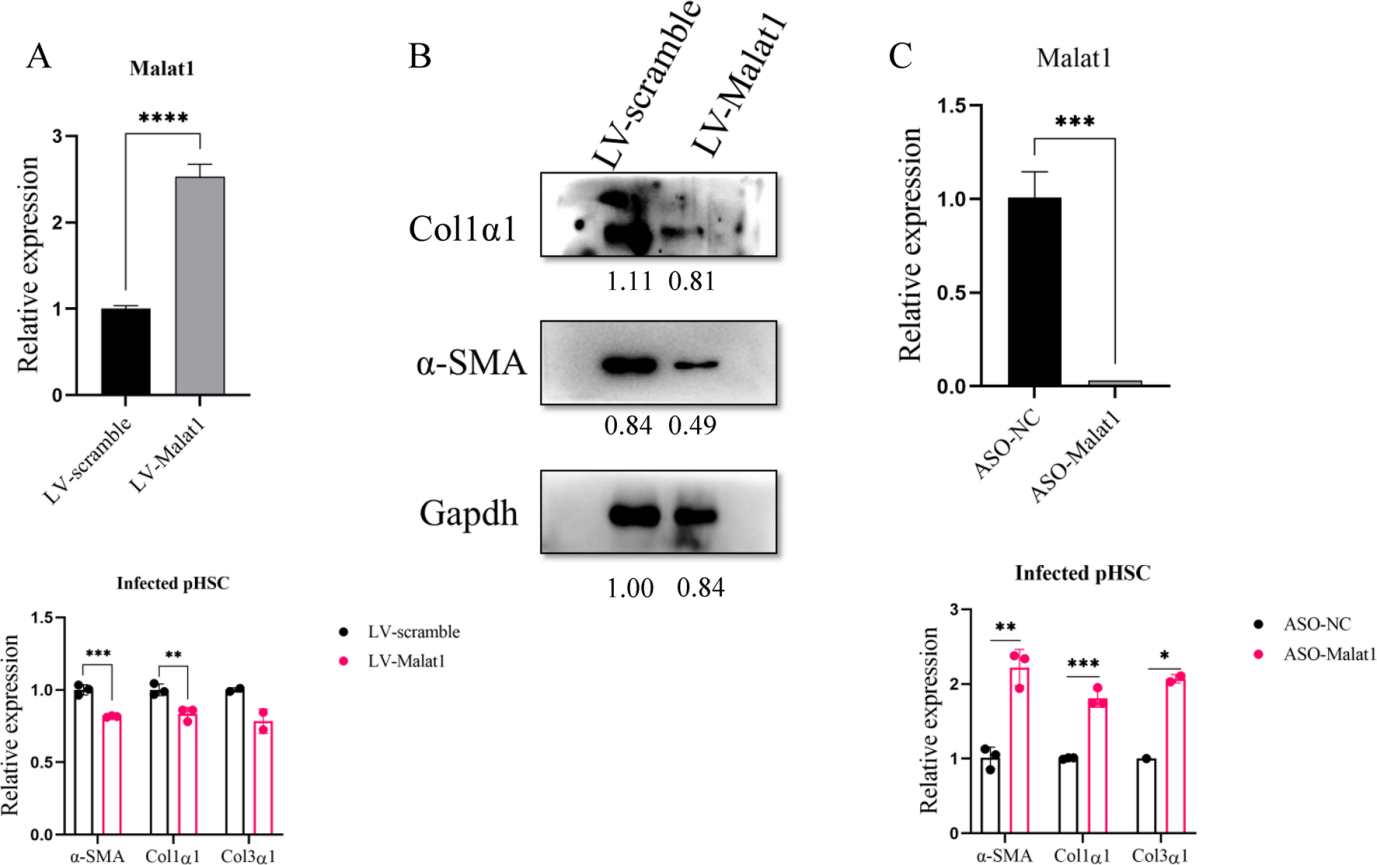
Forced Malat1 expression inhibited the expression of fibrosis-related genes in vitro. Primary HSCs isolated from infected mice were cultured in vitro for 24 h and then used for the gain- or loss-of-function assays. **A** Expression of Malat1 and fibrosis-related genes detected by qPCR after infection with LV-Malat1 and LV-Scramble for 48 h (*n* = 3). **B** Protein levels of α-SMA and Col1α1 were detected in the Malat1 overexpression primary HSCs by Western blot. GAPDH was used as an internal control. **C** Expressions of Malat1 and fibrosis-related genes detected by qPCR in the HSCs transfected with ASO-Malat1 and ASO-NC for 48 h (*n* = 3). * *p* < 0.05, ** *p* < 0.01, *** *p* < 0.001, **** p < 0.0001

To explore the biological effects of Malat1 on schistosomiasis HF, we constructed a Malat1 lentivirus for overexpression and designed the compound of ASO-Malat1 for knockdown in the infected primary HSCs, respectively. The primary HSCs isolated from infected mice at day 49 were transfected with Malat1 lentivirus, which significantly elevated the expression of Malat1 in the transfected HSC compared to the scramble control, leading to a reduction in the expression of fibrosis-related genes encoding α-SMA and Col1α1 (Fig. 2A). Protein levels of α-SMA and Col1α1 were also reduced in the transfected HSC with Malat1 lentivirus detected by Western blot (Fig. 2B). Furthermore, the infected primary HSCs at day 49 transfected with ASO-Malat1 markedly silenced the Malat1 expression, leading to an increase in the expression of the fibrosis-related genes (Fig. 2C). These findings suggested a negative regulation of fibrosis-related gene expression in HSC by Malat1 lncRNA in schistosomiasis HF.

### Interaction of Malat1 with miRNA-96.

Studies have shown that lncRNAs can interact with DNA, RNA or protein to regulate gene expression at various levels [23–25].One of the classic pathways is of a competitive endogenous RNA adsorbing miRNA molecules to regulate gene expression [26]. We have identified several host miRNAs that regulate schistosomiasis HF [10, 11, 27]. Therefore, we investigated whether the Malat1-mediated suppression of fibrosis-related genes in the schistosomiasis HF occurs through interaction with miRNAs.

**Fig. 3:**
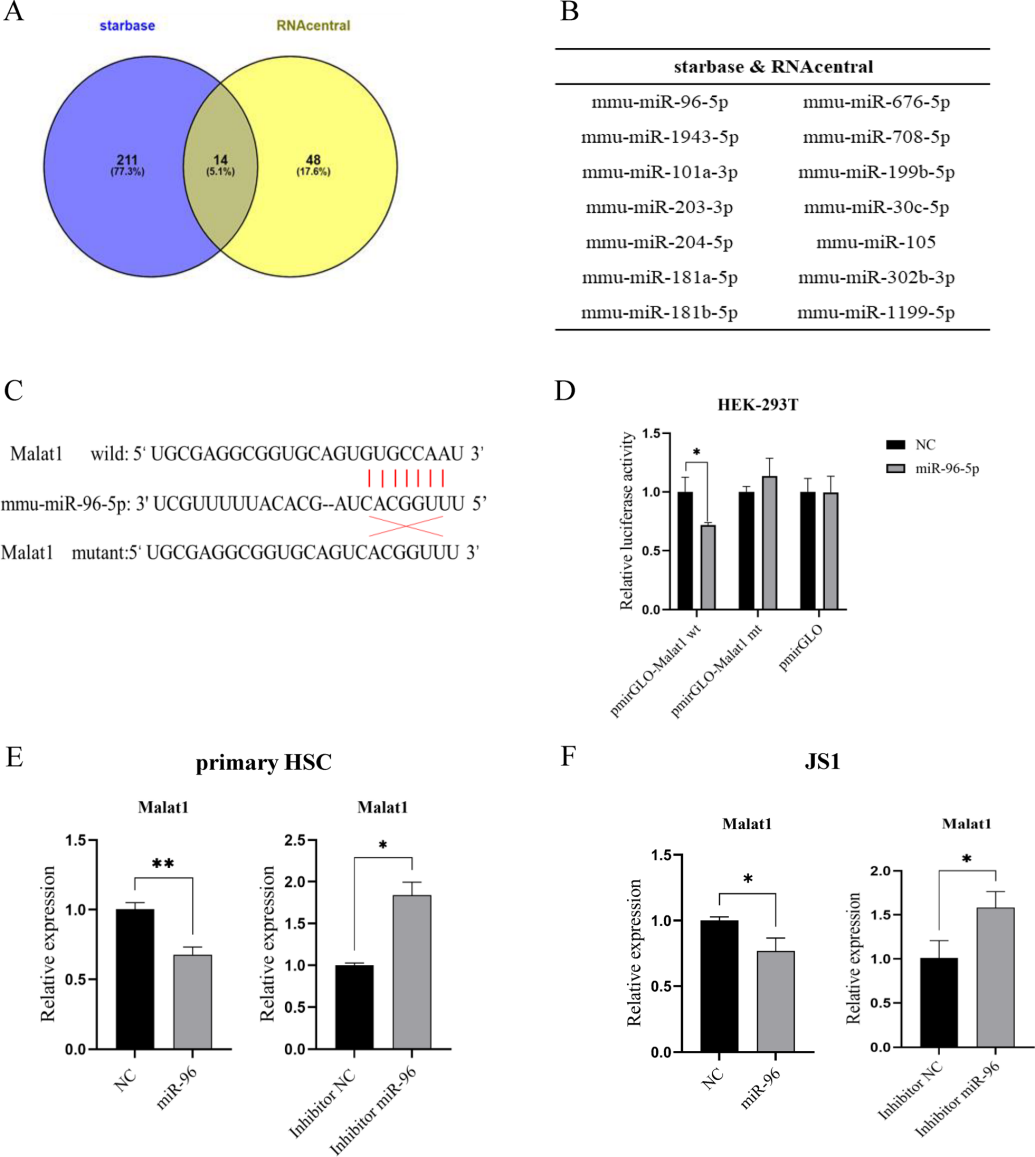
Interaction of Malat1 with miRNA-96. Common target miRNAs predicted by Starbase and RNAcentral are shown in Venn diagrams (**A**) and names of these miRNAs are listed in (**B**). **C** Predicted wild-type and mutant-binding site between Malat1 and miRNA-96. **D** Luciferase reporter assays were done to verify the validity of the predicted binding sites of Malat1 and miRNA-96 in HEK293T cells. Malat1 expression was evaluated by qPCR in primary HSCs (**E**) and JS1 (**F**) transfected with miRNA-96 mimics or inhibitor miRNA-96 for 48 h. * *p* < 0.05, ** *p* < 0.01

We first performed a bioinformatics analysis using the online databases Starbase [28] and RNAcentral (https://rnacentral.org/). Venn diagram analysis of the miRNAs predicted by the two databases generated 14 common miRNAs (Fig. 3A, B). It was reported that miRNA-96 regulated schistosoma-associated liver fibrosis by targeting Smad7, an important inhibitor of TGF-β1 signaling [11]. Moreover, schistosome infection significantly induced the expression of miRNA-96 [11], which was opposite to that of Malat1, implying the Malat1-mediated inhibition of hepatic fibrosis might be through miRNA-96. To investigate potential interaction of Malat1 with miRNA-96, we constructed both wild type (wt) and mutation type (mt) Malat1 luciferase reporter plasmids based on the predicted binding site between Malat1 and miRNA-96 (Fig. 3C) and transfected HEK-293 T cells with both plasmids as well as miRNA-96 mimics or NC mimics. The results showed that the relative luciferase activities in the cells transfected with wt Malat1 and miRNA-96 mimics were significantly reduced compared to those with NC mimics, while the relative luciferase activities were unaffected in the mt Malat1 group (Fig. 3D). Additionally, transfection of primary HSC isolated from normal mice with miRNA-96 mimics led to a decrease of Malat1 levels (Fig. 3E), while transfection with inhibitor miRNA-96 increased the Malat1 expression (Fig. 3E). Similar results were obtained in the murine HSC cell line JS1 (Fig. 3F). To further determine the adsorbing relationship between Malat1 and miR-96, we constructed the binding sequence and its mutant sequence and inserted them into pcDNA plasmid. We then transfected them into JS1 and human HSC cell line LX-2, respectively. qPCR detection showed that the expression levels of miRNA-96 decreased significantly in the WT group but did not change in the MT group in either cell (Figure S2), suggesting that Malat1 is involved in regulating the expression of miR-96 in the HSC cell lines.

### Localization of intracellular interactions of Malat1 and miRNA-96.

**Fig. 4:**
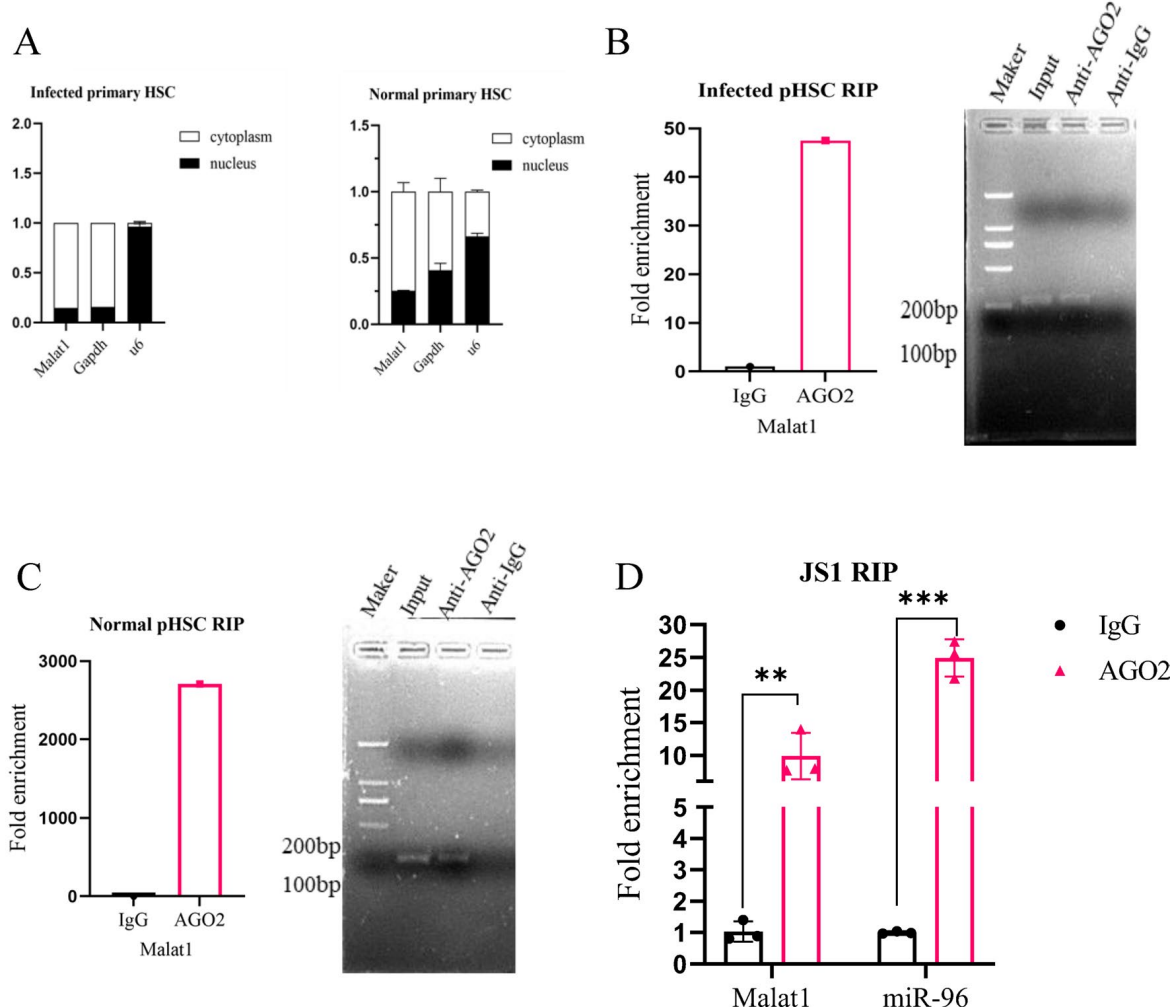
Localization of intracellular interactions of Matat1 and miRNA-96. (**A**) Nuclear cytoplasm fractionation was done to determine subcellular accumulation of Malat1 in infected and normal primary HSCs. **B**–**C** In both infected and normal primary HSCs, Ago2-RIP assay followed by qPCR and agarose gel electrophoresis was used to measure the enrichment of Malat1 in RISC. **D** Malat1 and miRNA-96 expression was validated in RISC via anti-Ago2 RIP assays in JS1. *p < 0.05, **p < 0.01, ***p < 0.001

As described above, the Malat1-mediated suppression of schistosomiasis HF likely occurs through interaction with miRNA-96 located in the cytoplasm. Thus, defining the subcellular localization of the Malat1 lncRNA is critical for elucidating its molecular mechanism. We isolated primary HSCs from both infected and uninfected mice. Subcellular fractionation followed by qPCR showed that Malat1 was mainly located in the cytoplasm of primary HSCs from both normal and infected HSCs (Fig. 4A), indicating that *S. japonicum* infection does not change the localization of Malat1. Moreover, miRNAs can bind their target gene to regulate RNA expression through RNA-induced silencing complex (RISC) in an Ago2-dependent manner. To further identify the interactive binding of Malat1 with miRNA-96, the antibody against Ago2 was used to conduct RIP assay. Compared with negative IgG-RIP control, Malat1 was significantly enriched in Ago2-RIP detected by both qPCR and agarose gel electrophoresis in both infected and normal primary HSCs (Fig. 4B, C). Notably, both Malat1 and miRNA-96 were also significantly enriched in the Ago2-RIP of JS1 cells (Fig. 4D). Thus, all data indicated the presence of Malat1 in cytoplasm and RISC complex.

### Malat1 exerts its effect through Malat1/miRNA-96/Smad7 axis

**Fig. 5:**
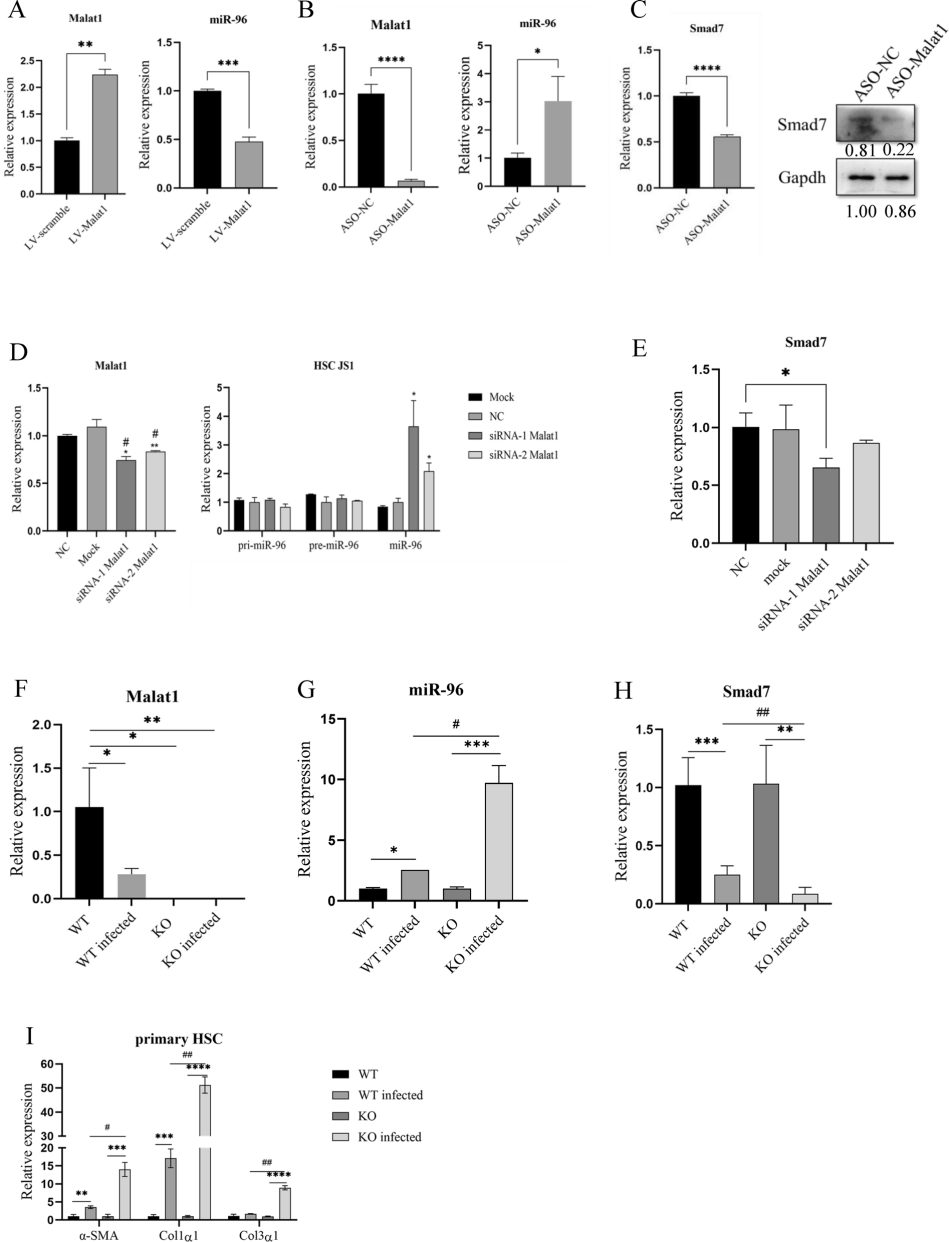
Malat1 exerts its effect through the Malat1/miRNA-96/Smad7 axis. **A**–**B** Malat1 regulated miRNA-96 expression in infected primary HSCs cultured in vitro through infection with LV-Malat1 or transfection with ASO-Malat1 by qPCR validation. **C** qPCR and WB assays used to verify the mRNA and protein expression levels of Smad7 after Malat1 knock down in infected primary HSCs. **D** Interference effectiveness of two siRNA-Malat1s and their effects on the expression of pri-miRNA-96, pre-miRNA-96 and miRNA-96 in JS1 cells detected by qPCR. **E** qPCR detection of the expression levels of Smad7 after Malat1 knock down in JS1. **F**–**H** qPCR detection of the expressions of Malat1, miRNA-96 and Smad7 in isolated primary HSCs of infected and uninfected wild-type and Malat1-KO mice. **I** Expression of fibrosis-related genes in isolated primary HSCs of wild-type and Malat1-KO mice. *p < 0.05, **p < 0.01, ***p < 0.001, ****p < 0.0001, #*p* < 0.05, ##*p* < 0.01

Our published data showed that *S. japonicum* infection significantly upregulated miRNA-96 expression, promoting schistosomiasis HF in mice by suppressing Smad7 [11]. Therefore, we investigated whether the Malat1-mediated suppression of schistosomiasis HF occurs through the Malat1/miRNA-96/Smad7 axis. qPCR data showed that overexpression of Malat1 by lentivirus significantly reduced miRNA-96 in infected primary HSCs in vitro (Fig. 5A). Inhibition of Malat1 markedly elevated miRNA-96 (Fig. 5B), thereby reducing Smad7 expression detected by both qPCR and Western blot (Fig. 5C). Furthermore, two siRNAs for Malat1 were transfected into JS1 cells, and the detection of mRNA expression indicated both effectively reduced Malat1 level (Fig. 5D, left), leading to elevated miRNA-96 levels (Fig. 5D, right), and reduced Smad7 expression (Fig. 5E). Notably, reduction of Malat1 did not affect pri-miR-96 and pre-miR-96 expression (Fig. 5D, right).

Next, we investigated the Malat1/miRNA-96/Smad7 axis in primary HSCs isolated from wild-type and Malat1-KO mice infected or uninfected with *S. japonicum*. The Malat1-KO mice had complete knockout of Malat1 via CRISPR/Cas9 technology. We showed that in the wild-type HSCs, *S. japonicum* infection dramatically reduced the Malat1 levels, while in the KO HSCs, the Malat1 was undetectable (Fig. 5F), indicating complete deletion of the Malat1 in the mice. We then examined the expressions of miRNA-96 and Smad7. In the Malat1-KO infected mice, miRNA-96 expression was significantly upregulated compared to wild-type infected and Malat1-KO uninfected mice (Fig. 5G); correspondingly, Smad7 expression was significantly reduced (Fig. 5H). In addition, the profibrogenic genes of α-SMA, Col1α1 and Col3α1 were highly expressed in both wild-type and Malat1-KO infected mice, but their expression levels were significantly increased in Malat1-KO infected compared to wild-type infected mice (Fig. 5I). Taken together, these data revealed that in the infected mice, knockout of Malat1 gene upregulated miRNA-96 expression, leading to a reduction of Smad7 expression, indicating that the Malat1-mediated suppression of the schistosomiasis HF occurs through the Malat1/miRNA-96/Smad7 axis.

### Effect of knockout of Malat1 or restored its expression on schistosomiasis HF

**Fig. 6:**
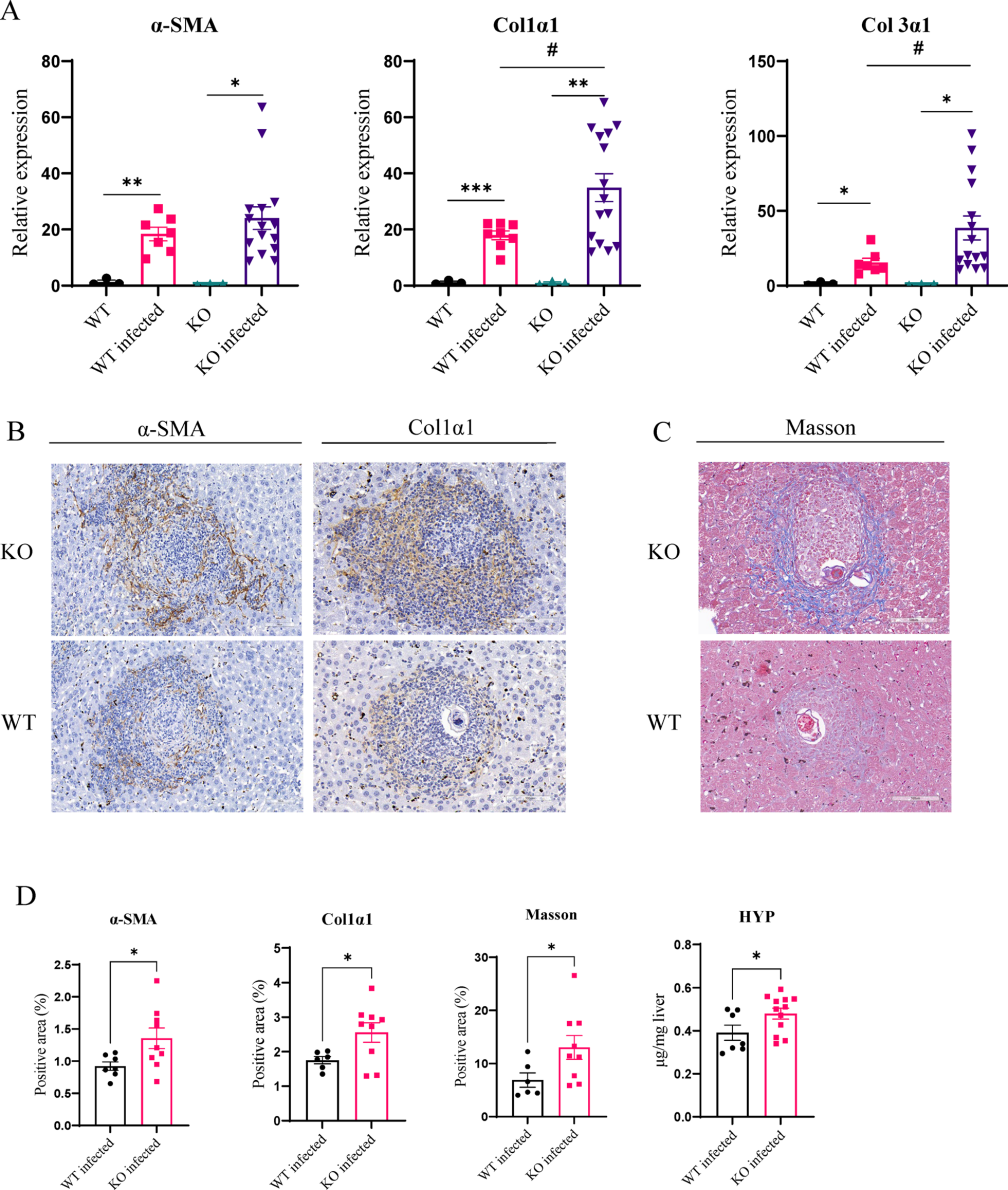
Malat1-KO mice aggravated liver fibrosis caused by *Schistosoma japonicum* infection. **A** qPCR detection of the expression of fibrosis-related genes in the liver tissues of infected or uninfected wild-type and Malat1-KO mice. The IHC (**B**) and Masson’s trichrome staining (**C**) analysis of liver tissue sections in the wild-type and Malat1-KO infected mice. **D** Positive area and HYP determined from IHC and Masson's trichrome staining of liver tissues by image J software and Hydroxyproline Content Assay Kit. *p < 0.05, **p < 0.01, ***p < 0.001, #*p* < 0.05

We next examined the role of Malat1 in the progression of schistosomiasis HF. The Malat1-KO or wild-type mice were infected with *S. japonicum* cercariae, and the liver tissues were collected at day 49 post-infection for qPCR, IHC and collagen deposition analyses. As shown in Fig. 6A, compared to wild-type mice, knocking out Malat1 increased mRNA expression levels of α-SMA, Col1α1 and Col3α1 caused by *S. japonicum* infection. Additionally, IHC and Masson’s trichrome staining results indicated increased expression of α-SMA and Col1α1 proteins and more collagen deposited in the Malat1-KO infected mice compared to the wild-type infected mice (Fig. 6B, C). Positive area analysis results of IHC and Masson’s trichrome staining are shown in Fig. 6D. Furthermore, the hydroxyproline content was significantly elevated in the Malat1-KO infected mice (Fig. 6D). In addition, the number of adult worms and eggs trapped in liver tissues showed no statistically significant difference between groups, but serum alanine aminotransferase (ALT) levels were significantly higher in knockout mice compared to wild-type mice (Figure S3), suggesting more severe liver injury. These data suggest that knockout of Malat1 aggravates schistosomiasis HF.

**Fig. 7:**
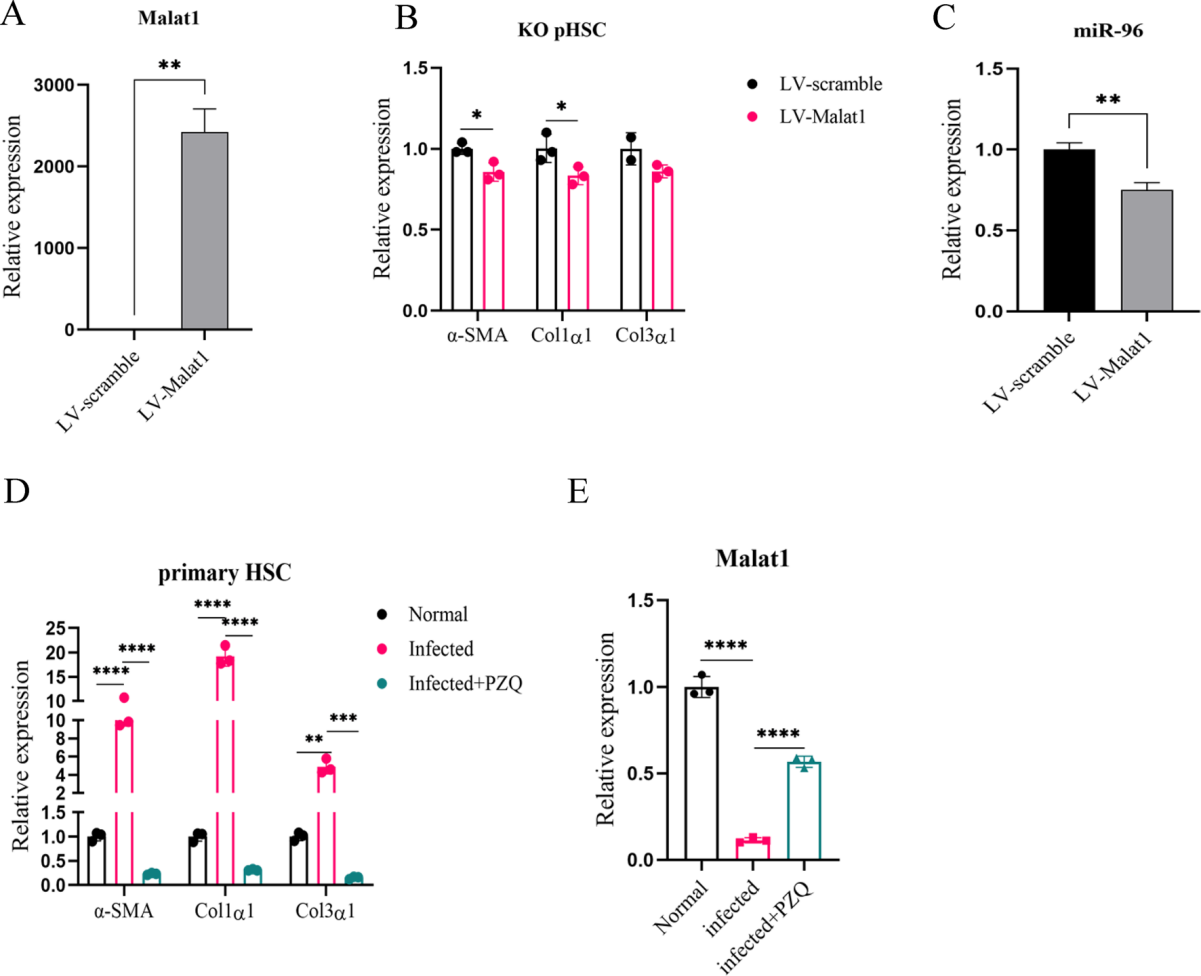
Effect of knockout of Malat1 or restoration of its expression on schistosomiasis HF. Primary HSCs were isolated from Malat1-KO mice cultured in vitro and then infected with LV-Malat1 and LV-Scramble. The expression of Malat1 (**A**) and fibrosis-related genes (**B**) and miRNA-96 (**C**) was measured by qPCR (*n* = 3). Infected mice were treated with PZQ at day 42, and primary HSCs were isolated at day 56 for detection of expression of Col1α1, Col3α1, α-SMA (**D**) and Malat1 (**E**) by qPCR. *p < 0.05, **p < 0.01, ***p < 0.001, ****p < 0.0001

We next examined the role of restored expression of Malat1 in the regulation of schistosomiasis HF. HSCs were isolated from the infected Malat1-KO mice and cultured in vitro. Restored expression of Malat1 by lentivirus reduced expression of α-SMA and Col1α1 at mRNA levels (Fig. 7A, B), leading to a reduction in expression of miRNA-96 (Fig. 7C). Praziquantel (PZQ) is a safe and effective anti-schistosome drug, but its therapeutic effect on hepatic fibrosis of schistosomiasis japonica has also been reported [29]. Both anti-parasite and anti-fibrosis treatment of PZQ significantly reduced expression of profibrogenic genes. To evaluate the relationship between Malat1 and profibrogenic gene expression in infected mice treated with PZQ, we started treatment with PZQ at day 42 post-infection and isolated HSCs at day 56 for analysis of the profibrogenic genes. We showed that PZQ treatment significantly reduced expression of genes encoding α-SMA, Col1α1 and Col3α1 (Fig. 7D). Interestingly, PZQ treatment dramatically elevated the expression of Malat1 compared to the untreated infected mice (Fig. 7E), indicating that Malat1 expression in the infected mice is sensitive to PZQ chemotherapy. Thus, these data indicated the negative correlation of Malat1 expression with the expression of profibrogenic genes in infected mice, including those treated with PZQ.

## Discussion

The major pathological change in *S. japonicum* infection is liver fibrosis caused by immunopathological reactions against parasite eggs trapped in liver tissues, where they secrete various components triggering inflammatory immune responses and the formation of granulomas [30]. Schistosomiasis HF is the primary cause of morbidity and mortality from the disease. The mechanism underlying the fibrosis is complex, involving activation of HSCs and TGF-β/SMAD signaling [31]. Our previous studies showed that *S. japonicum* infection induces the dysregulation of certain miRNAs concomitant with hepatic fibrosis. For example, parasite infection-mediated upregulation of both miRNA-21 and miRNA-96 induces hepatic fibrosis by targeting SMAD7, thereby relieving the inhibitory effects of SMAD7 on the TGF-β/SMAD pathway [10, 11]. In addition, schistosome-derived miRNAs, such as sja-miRNA-2162 and sja-miRNA-1, are involved in the progression of schistosomiasis HF during infection [32, 33]. Recently, lncRNAs have been reported to be involved in regulation of various diseases such as cancers and fibrosis [34]. However, it remains unclear whether lncRNAs regulate the progression of schistosomiasis HF. Although many lncRNAs have been identified in the schistosome genome [21, 35, 36], few studies have addressed their role in hepatic fibrosis. However, schistosome infection can induce dysregulation of host lncRNAs [37, 38], but their role in schistosomiasis HF has not been investigated. In this study, we identified altered expression of lncRNA profiles in HSCs from *S. japonicum*-infected mice and showed that Malat1 was significantly downregulated in the HSCs of infected mice. Forced expression of Malat1 in infected primary HSC resulted in reduction in the expression of profibrogenic genes, while silencing the Malat1 gene had the opposite effect, displaying a negative correlation between Malat1 expression and the expression of profibrogenic genes. Furthermore, we demonstrated that the negative regulation occurs through the Malat1/miRNA-96/Smad7 axis. It has been reported that schistosome infection significantly upregulated miRNA-96 expression, which elicits hepatic fibrosis by targeting SMAD7 [11]. Here, we showed that inhibition of Malat1 by ASO obviously increased miRNA-96 and thereby reduced the expression of Smad7, relieving the inhibitory effects of SMAD7 on the TGF-β/SMAD pathway and thereby elevating the expression of fibrosis-related genes. Notably, Malat1-KO mice infected with *S. japonicum* exhibited more severe fibrosis than wild-type infected mice, as manifested by higher expression of miRNA-96 and a lower level of Smad7. Interestingly, Malat1 expression in the infected mice is sensitive to PZQ treatment: PZQ treatment dramatically elevated the expression of Malat1. Therefore, our data suggest Malat1 is not only a potential therapeutic target for schistosoma-associated liver fibrosis but also a biomarker for assessing the response to PZQ chemotherapy. Malat1 is a well-studied lncRNA involved in the regulation of multiple diseases. Malat1 has been reported to acts as an oncogene in various cancers. For example, Malat1 can promote esophageal squamous cell carcinoma (ESCC) by modifying the ATM-CHK2 pathway [39]. However, the role of Malat1 in cancer is controversial. Cao et al. reported that Malat1 expression was significantly decreased in glioma specimens, and overexpression of Malat1 suppressed glioma cell viability through the Malat1/miRNA-155/FBXW7 axis [40]; Han et al. also demonstrated the tumor-suppressive function of MALAT1 in glioma cells by downregulation of MMP2 and inactivation of ERK/MAPK signaling [41]. Additionally, Kim et al. proved Malat1 suppressed breast cancer metastasis [42]. These data suggest that the role of the Malat1 in cancer depends on the type of cancer and its mechanism.

Malat1 is also involved in several fibrotic diseases. In diabetic nephropathy, upregulation of Malat1 promotes renal fibrosis by targeting the miRNA-2355-3p, while in an experimental post-infarct myocardium mouse model, Malat1 mediates cardiac fibrosis by regulating TGF‐β1 activity via miRNA-145 [18, 19]. In addition, melatonin administration significantly reduced collagen production by inhibiting Malat1/miRNA-141-mediated NLRP3 inflammasome and TGF-β1/SMADs signaling [43]. In arsenite-induced liver fibrosis, elevated Malat1 is involved in the activation of hepatic stellate cells via miRNA-26b [44]. In these studies, Malat1 was upregulated and promoted fibrosis via an endogenous sponge of different miRNAs related to fibrosis [20, 44, 45]. The role of Malat1 in fibrotic diseases is also likely dependent on the type of fibrotic diseases and its mechanisms. In this study, we showed that in a well-studied murine model of human schistosomiasis, Malat1 was dramatically downregulated, differing from other fibrotic disease models. This could be due to the complex mechanism of fibrosis induced by schistosome infection. The schistosome parasite contains more than 10,000 putative encoding genes, making the mechanism of schistosome-induced liver fibrosis more complex and distinct from other fibrosis models, such as the CCL4 chemically induced hepatic fibrosis model [46]. For example, the regulators of schistosomiasis HF are complicated. In addition to TGF-β1, which is the most important regulator for various tissue fibrosis, IL-13 is a critical profibrotic factor in schistosomiasis-associated liver fibrogenesis, exerting its effect independent of TGF-β1 [9]. Furthermore, lncRNAs can sponge miRNAs to regulate gene expression and the pathogenesis of the diseases. Thus, the involved miRNA should also play a crucial role in the regulatory process. Our previous data revealed that schistosome infection induces expression of miRNA-96 and exerts its pro-fibrotic effect by targeting SMAD7 [11]. Here, we demonstrated that Malat1 is the target of miRNA-96 and functions as an endogenous sponge to regulate the functional availability of miRNA-96. We further demonstrated this in the Malat1-KO infected mice, showing that Malat1 knockout significantly upregulated miRNA-96 expression, leading to reduced Smad7 expression and elevated expression of profibrogenic genes α-SMA, Col1α1 and Col3α1, resulting in a more severe schistosomiasis HF. Restoring expression of Malat1 in HSCs from infected Malat1-KO mice reduced the expression of profibrogenic genes. Thus, we can summarize the mechanism as schistosome infection induces downregulation of Malat1 expression, increasing the miRNA-96 expression by reducing Malat1-mediated endogenous sponging, and elevated miRNA-96 promotes schistosomiasis HF by targeting Smad7.

## Conclusions

This study demonstrates that *S. japonicum* infection-induced downregulation of Malat1 expression contributes to schistosomiasis HF via the Malat1/miR-96/Smad7 axis. Elevated Malat1 expression in infected HSCs reduces fibrosis by downregulating miR-96 and upregulating Smad7, thus providing a novel therapeutic target for schistosomiasis hepatic fibrosis. Furthermore, Malat1 can serve as a biomarker for assessing the response to PZQ treatment, offering potential strategies for intervention of schistosomiasis HF.

## Supplementary Information

Additional file 1: Figure. 1. Malat1 expression was downregulated in the infected liver. The expression levels of Malat1 in the whole liver slides was evaluated by Digoxin-labeled probe and in situ hybridization. (A) Normal liver slide. (B) Infected liver slide. (C) Average optical density histogram. The arrows indicate positive signal of hybridization. * p < 0.05.

Additional file 2: Figure. 2. Expression levels of miRNA-96. JS1 and LX-2 cells were transfected with empty or correspondinglplasmids which contain the wild-type or mutant binding site between Malat1 and miRNA-96; 48 h later, the expression levels of binding site and miRNA-96 were detected by qPCR. *p < 0.05, **p < 0.01, ***p < 0.001, ****p < 0.0001.

Additional file 3: Figure. 3. Serum ALT levels and number of parasites and eggs in different group. (A) Serum was collected from WT and Malat1-KO mice with or without *Schistosoma japonicum* infection, and ALT was detected. Recovered parasites (B) and eggs (C) deposited in the liver were counted and compared. *p < 0.05, **p < 0.01.

Additional file 4: Table S1. Sequences of primers used for qPCR or PCR.

Additional file 5: Table S2. List of siRNAs, miRNA-96 mimics and inhibitor.

## Data Availability

No datasets were generated or analysed during the current study.

## Abbreviations

*S*. *japonicum*: *Schistosoma* *japonicum*
HSCs: Hepatic stellate cells
PZQ: Praziquantel
α-SMA: Alpha-smooth muscle actin
lncRNAs: Long non-coding RNAs
HF: Hepaticfibrosis
H&E: Hematoxylin–eosin
RT-qPCR: Quantitative real-time PCR
mean ± SD: Mean ± standard deviation
