# Supplementary figures and images for: Schistosoma japonicum infection-mediated downregulation of lncRNA Malat1 contributes to schistosomiasis hepatic fibrosis by the Malat1/miR-96/Smad7 pathway
Source: Parasit Vectors. 2024 Oct 3;17:413. doi: 10.1186/s13071-024-06499-9 (PMC11451255; doi:10.1186/s13071-024-06499-9)

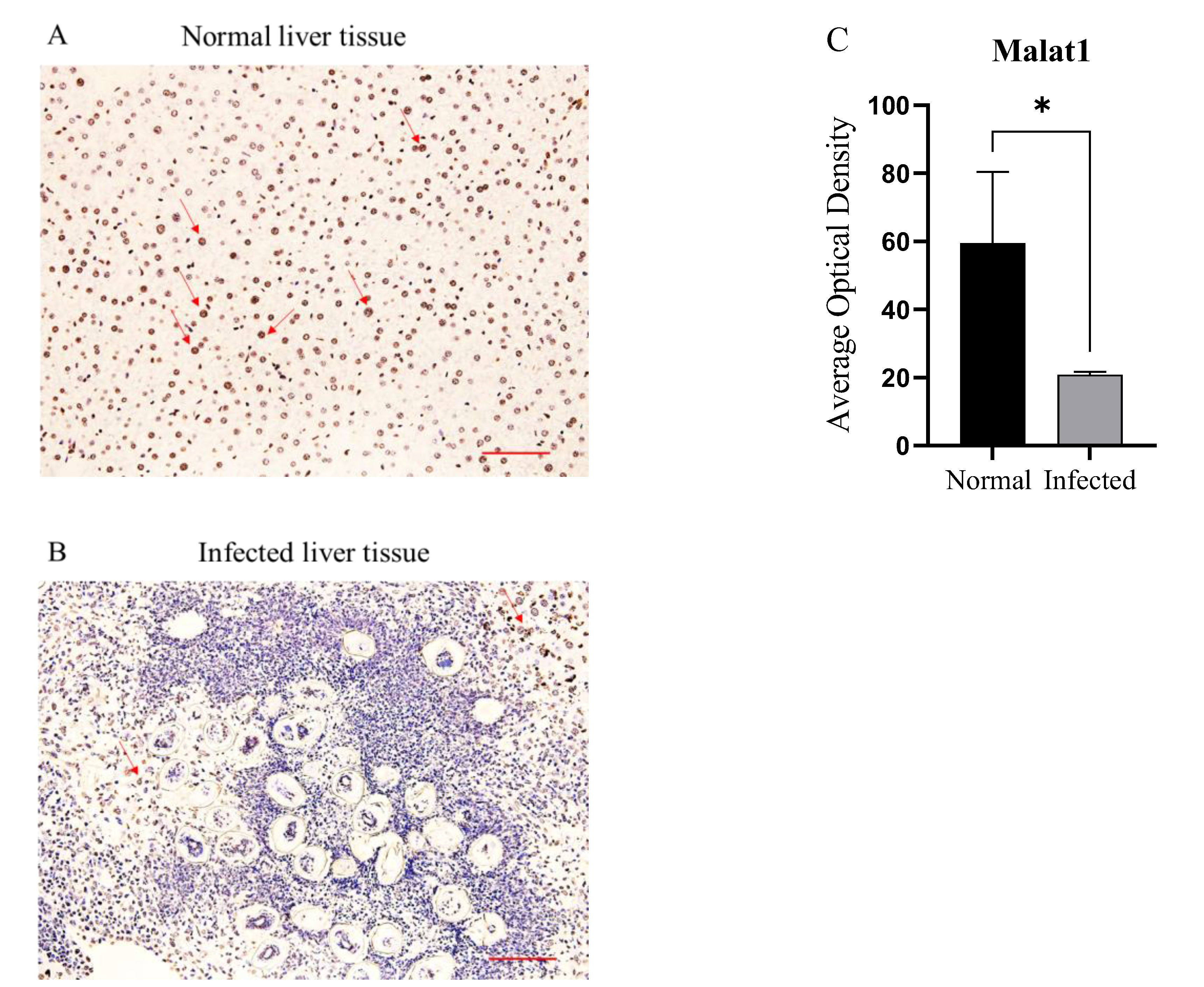

Supplement: Supplementary file 1 — Additional file 1: Figure. 1. Malat1 expression was downregulated in the infected liver. The expression levels of Malat1 in the whole liver slides was evaluated by Digoxin-labeled probe and in situ hybridization. (A) Normal liver slide. (B) Infected liver slide. (C) Average optical density histogram. The arrows indicate positive signal of hybridization. * p < 0.05. [file 13071_2024_6499_MOESM1_ESM.tiff]

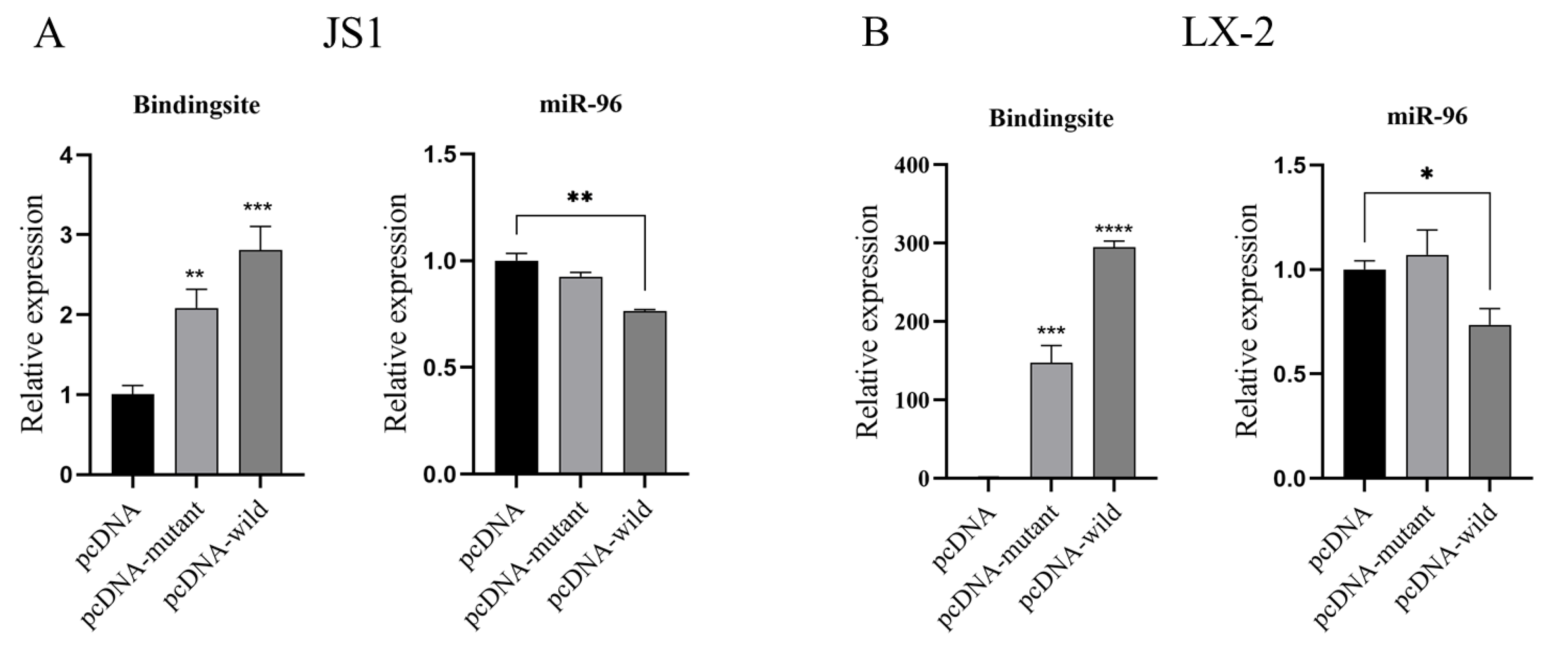

Supplement: Supplementary file 2 — Additional file 2: Figure. 2. Expression levels of miRNA-96. JS1 and LX-2 cells were transfected with empty or correspondinglplasmids which contain the wild-type or mutant binding site between Malat1 and miRNA-96; 48 h later, the expression levels of binding site and miRNA-96 were detected by qPCR. *p < 0.05, **p < 0.01, ***p < 0.001, ****p < 0.0001. [file 13071_2024_6499_MOESM2_ESM.tiff]

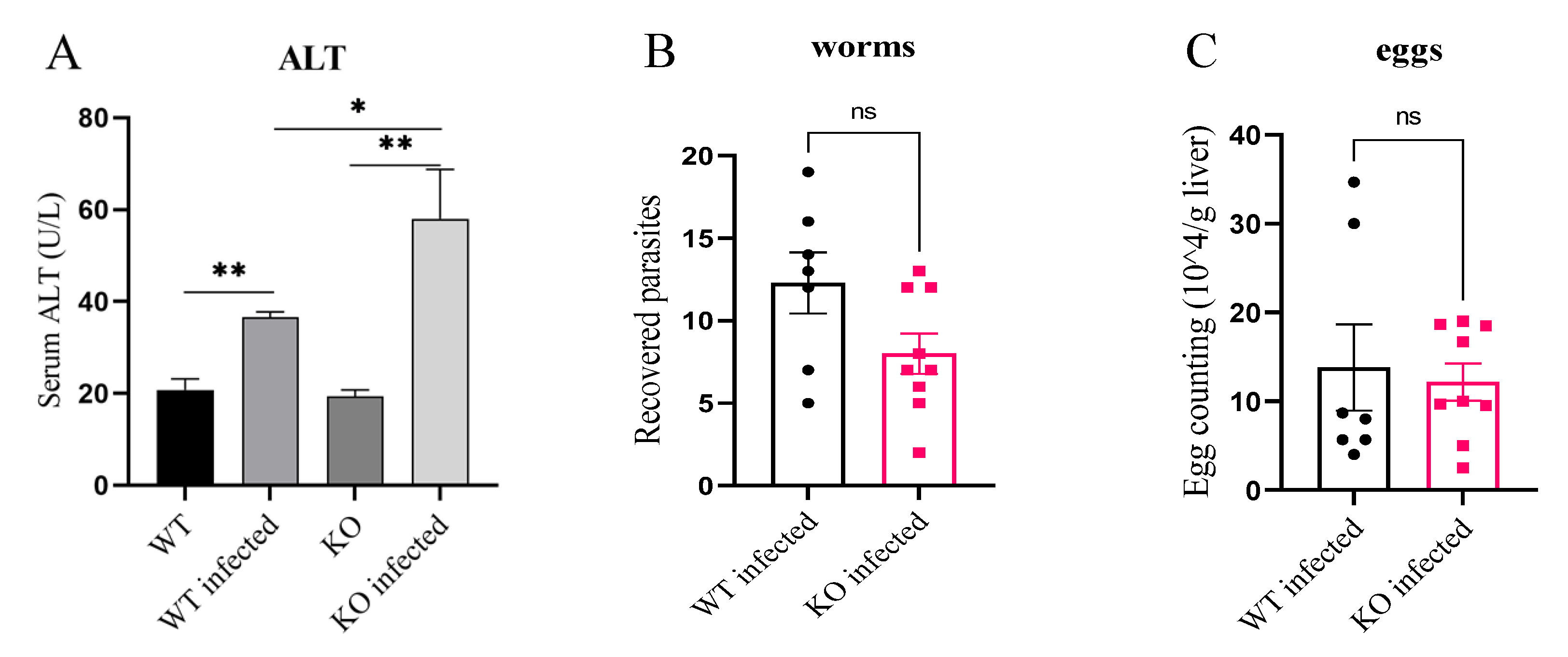

Supplement: Supplementary file 3 — Additional file 3: Figure. 3. Serum ALT levels and number of parasites and eggs in different group. (A) Serum was collected from WT and Malat1-KO mice with or without Schistosoma japonicum infection, and ALT was detected. Recovered parasites (B) and eggs (C) deposited in the liver were counted and compared. *p < 0.05, **p < 0.01. [file 13071_2024_6499_MOESM3_ESM.tiff]
