# Supplementary material for: Schistosoma japonicum infection-mediated downregulation of lncRNA Malat1 contributes to schistosomiasis hepatic fibrosis by the Malat1/miR-96/Smad7 pathway
Source: Parasit Vectors. 2024 Oct 3;17:413. doi: 10.1186/s13071-024-06499-9 (PMC11451255; doi:10.1186/s13071-024-06499-9)
Supplement: Supplementary file 4 — Additional file 4: Table S1. Sequences of primers used for qPCR or PCR. [file 13071_2024_6499_MOESM4_ESM.docx]

# Supplementary information:

Table S1. Sequences of primers for qPCR or PCR

| Gene names | Sequences (5'-3') |
| --- | --- |
| SMA qFP | GTCCCAGACATCAGGGAGTAA |
| SMA qRP | TCGGATACTTCAGCGTCAGGA |
| Col1α1 qFP | GACGCCATCAAGGTCTACTGC |
| Col1α1 qRP | GGAAGGTCAGCTGGATAGCG |
| Col3α1qFP | TCCCCTGGAATCTGTGAATC |
| Col3α1 qRP | TGAGTCGAATTGGGGAGAAT |
| Malat1 qFP | GAGTTGTAGGCTTCTGTGTA |
| Malat1 qRP | AGGCTTGTGGTAGGTCAT |
| Smad7 qFP | GGCCGGATCTCAGGCATTC |
| Smad7 qRP | TTGGGTATCTGGAGTAAGGAGG |
| Gapdh qFP | AGGTCGGTGTGAACGGATTTG |
| Gapdh qRP | TGTAGACCATGTAGTTGAGGTCA |
| Gas5 FP | GGATAACAGAGCGAGCGCAAT |
| Gas5 RP | CCAGCCAAATGAACAAGCATG |
| F630028O10Rik qFP | AAGACAAAGGCACCACTTCAATG |
| F630028O10Rik qRP | CCACCAGCAGTTTTCTAAAGGATG |
| MSTRG. 76741 qFP | cagacatgtttgtggatttaccc |
| MSTRG. 76741 qRP | cttgatagaaacggggatgg |
| MSTRG. 40774 qFP | agttgacaaggacagctggaa |
| MSTRG. 40774 qRP | catgagctcagtctcttgaagatt |
| ENSMUST00000145435 qFP | cccagggctcactcactct |
| ENSMUST00000145435 qRP | catgtccatagcaccctgag |
| MSTRG. 38136 qFP | AACTCAAGGACCCAGACC |
| MSTRG. 38136 qRP | AGGCAGGAACTTAAATAGGA |
| MSTRG. 67416 qFP | TCTAACCCACTGAGCCATCT |
| MSTRG. 67416 qRP | CTCCAAGCCATCCCTCTA |
| MSTRG. 69300 qFP | AGCCAGCAAGCGGTAAGT |
| MSTRG. 69300 qRP | AGCCAGGAGTGAGCCATT |
| U6 qFP | ATGGGTCGAAGTCGTAGCC |
| U6 qRP | TTCTCGGCGTCTTCTTTCTCG |
| pri-96 qFP | GGGCCATAAACAGAGCAGAG |
| pri-96 qRP | GGCAGTGAAAGGTGATCTGG |
| pre-96 qFP | TACCATCTGCTTGGCCGATT |
| pre-96 qRP | ACATGATTGCTCACAGCGGA |
| pc-BS qFP | AGCAACCAGTATAAAGTTATGGCAA |
| pc-BS qRP | AACCTGTCTGAGGCAAACGAA |
| pmir-malat1 FP1 | TCTAGTTGTTTAAACGAGCTCACTGATGAAAAGCTGCATATG |
| pmir-malat1 RP1 | CCTGCAGGTCGACTCTAGATGCAAACATTCAACAGCTCTAC |
| pmir-malat1 FP2 | UUUGGCACUAGCACAUUUUUGCUTGTTTCGTTTGCCTCAGACAG |
| pmir-malat1 RP2 | AGCAAAAATGTGCTAGTGCCAAAAGCCGCTCGATCCCACTCATC |
| pCDH-Malat1 FP | gattctagagctagcgaattcCAGGCATTCAGGCAGCGAGAGC |
| pCDH-Malat1 RP | gatcgcagatccttcgcggccgcTGAGAGATATTTAGTTTTTATTTCATAAAATCAAAGTATTC |
| miRNA-96 RT | GTCGTATCCAGTGCAGGGTCCGAGGTATTCGCACTGGATACGACAGCAAA |
| miRNA-96 qFP | GCGTTTGGCACTAGCACATT |
| miRNA-96 qRP | AGTGCAGGGTCCGAGGTATT |
