# Supplementary material for: Schistosoma japonicum infection-mediated downregulation of lncRNA Malat1 contributes to schistosomiasis hepatic fibrosis by the Malat1/miR-96/Smad7 pathway
Source: Parasit Vectors. 2024 Oct 3;17:413. doi: 10.1186/s13071-024-06499-9 (PMC11451255; doi:10.1186/s13071-024-06499-9)
Supplement: Supplementary file 5 — Additional file 5: Table S2. List of siRNAs, miRNA-96 mimics and inhibitor. [file 13071_2024_6499_MOESM5_ESM.docx]

**Supplementary information:**

Table S2. List of siRNAs, miRNA-96 mimics and inhibitor

| Gene names | Sense (5'-3') | Antisense (5'-3') |
| --- | --- | --- |
| Malat1 siRNA-1 | AAGCCTACATGATTAATGCCT | AGGCATTAATCATGTAGGCTT |
| Malat1 siRNA-2 | GCAGTTTAGGAGATTGTAAAG | CTTTACAATCTCCTAAACTGC |
| Negative control | UUCUCCGAACGUGUCACGUTT | ACGUGACACGUUCGGAGAATT |
| miRNA-96 | UUUGGCACUAGCACAUUUUUGCU | AGCAAAAATGTGCTAGTGCCAAA |
| miR-96-5p Inhibitor | AGCAAAAAUGUGCUAGUGCCAAA | / |
| NC Inhibitor | CAGUACUUUUGUGUAGUACAA | / |
